# Adaptation to developmental diet influences the response to selection on age at reproduction in the fruit fly

**DOI:** 10.1111/jeb.13425

**Published:** 2019-02-27

**Authors:** Christina M. May, Joost van den Heuvel, Agnieszka Doroszuk, Katja M. Hoedjes, Thomas Flatt, Bas J. Zwaan

**Affiliations:** ^1^ Laboratory of Genetics Wageningen University Wageningen the Netherlands; ^2^ Institute for Cell and Molecular Biosciences Newcastle University Newcastle upon Tyne UK; ^3^ Rijk Zwaan Hague the Netherlands; ^4^ Department of Ecology and Evolution University of Lausanne Lausanne Switzerland; ^5^ Department of Biology University of Fribourg Fribourg Switzerland

**Keywords:** ageing, experimental evolution, life‐history evolution, phenotypic plasticity

## Abstract

Experimental evolution (EE) is a powerful tool for addressing how environmental factors influence life‐history evolution. While in nature different selection pressures experienced across the lifespan shape life histories, EE studies typically apply selection pressures one at a time. Here, we assess the consequences of adaptation to three different developmental diets in combination with classical selection for early or late reproduction in the fruit fly *Drosophila melanogaster*. We find that the response to each selection pressure is similar to that observed when they are applied independently, but the overall magnitude of the response depends on the selection regime experienced in the other life stage. For example, adaptation to increased age at reproduction increased lifespan across all diets; however, the extent of the increase was dependent on the dietary selection regime. Similarly, adaptation to a lower calorie developmental diet led to faster development and decreased adult weight, but the magnitude of the response was dependent on the age‐at‐reproduction selection regime. Given that multiple selection pressures are prevalent in nature, our findings suggest that trade‐offs should be considered not only among traits within an organism, but also among adaptive responses to different—sometimes conflicting—selection pressures, including across life stages.

## 1 INTRODUCTION

One of the central tenets of life‐history evolution is that individuals cannot simultaneously optimize all fitness‐related traits due to constraints (Roff, 1992, 2001; Stearns, 1992). These constraints can emerge because individuals have limited resources at their disposal and must make allocation decisions between competing functions (physiological constraints; Van Noordwijk & de Jong, 1986; de Jong & van Noordwijk, 1992) or because traits have a shared genetic basis (genetic constraints). Such constraints can lead to trade‐offs between traits, such that an increase in one trait comes at the expense of another (Stearns, 1992). For instance, the classical theories of ageing: the antagonistic pleiotropy (Williams, 1957) and disposable soma theories (Kirkwood, 1977) explain the evolution of ageing by genetic and physiological trade‐offs between survival and reproduction.

Experimental evolution (EE) is a powerful approach for understanding how life histories and trade‐offs evolve in response to specific environments. EE allows the experimenter to impose carefully controlled selective conditions in the laboratory and then observe evolutionary responses in real time (Kawecki et al., 2012). Two areas in which EE studies have been applied to great effect are in understanding how available nutrition influences life‐history evolution (Baldal, Brakefield, & Zwaan, 2006; Bubliy & Loeschcke, 2005; Chippindale, Chu, & Rose, 1996; Kolss, Vijendravarma, Schwaller, & Kawecki, 2009; Kristensen, Overgaard, Loeschcke, & Mayntz, 2011; Leftwich, Nash, Friend, & Chapman, 2016; Zajitschek et al., 2016) and in testing the classical theories of the evolution of ageing (Luckinbill, Arking, Clare, Cirocco, & Buck, 1984; Partridge & Fowler, 1992; Rose, 1984).

Experimental evolution studies manipulating available nutrition have identified several correlated changes in life‐history traits, with the magnitude and direction of the response depending on whether the dietary manipulation is applied during development or in adulthood. Adaptation to low resource availability during development typically results in decreased adult weight (Kolss et al., 2009; Kristensen et al., 2011), faster development (Kolss et al., 2009; Leftwich et al., 2016) and lower fecundity (Kolss et al., 2009), while effects on lifespan are small or absent (Kolss et al., 2009). In contrast, adaptation to low resource availability or starvation resistance during adulthood leads to slower development, increased lipid accumulation, larger adult size, increased lifespan and increased male fitness (Chippindale et al., 1996; Bubliy & Loeschcke, 2005; Baldal et al., 2006; Zajitschek et al., 2016, but see Hoffmann, Hallas, Anderson, & Telonis‐Scott, 2005).

EE studies testing the classical theories of ageing have applied selection for later ages at reproduction and show that increased lifespan can reliably evolve (Luckinbill et al., 1984; Partridge & Fowler, 1992; Rose, 1984). In most cases, decreased early or life‐long fecundity is observed as a correlate of lifespan extension, suggesting a trade‐off between lifespan extension and fecundity, as predicted by the disposable soma theory (Kirkwood & Holliday, 1979; Kirkwood & Rose, 1991; Zwaan, 1999).

Notably, the experiments described above each address the life‐history consequences of adaptation within a single life stage and to a single selection pressure (variation in diet or selection on increased age at reproduction). However, in nature, individuals will need to cope with multiple, potentially conflicting selection pressures (e.g., Lankau, 2007; Tarwater & Beissinger, 2013) experienced at different stages across the lifespan. Thus, they must balance the relative costs and benefits of adaptation and resource allocation made at one life stage with those at other stages (reviewed in Schluter, Price, & Rowe, 1991). Indeed, EE studies applying more than one selection pressure within a single life stage reveal that the responses to multiple selection pressures tend to be interdependent (Bochdanovits & Jong, 2003; Davidowitz, Roff, & Nijhout, 2016), yet also—despite constraining correlations among traits—there is potential for independent evolutionary change (Beldade & Brakefield, 2002; Frankino, Zwaan, Stern, & Brakefield, 2005). However, there has been little emphasis on how multiple selection pressures influence life histories as a whole. In particular, to the best of our knowledge, no study to date has combined two selection regimes experienced at different stages across an organism's lifespan.

**Figure 1:**
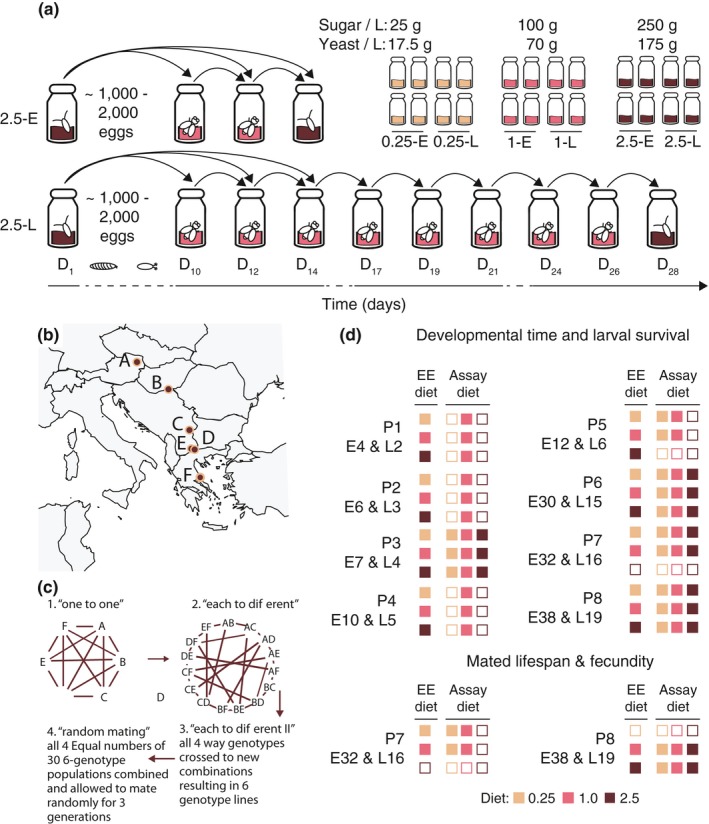
Experimental overview. (a) Experimental evolution design. Four replicate populations were established per combination of larval diet and age at reproduction (4 replicate lines * 3 larval diets * 2 ages at reproduction = 24 lines in total). The main panel traces a generation of EE for a single 2.5SY‐E (top) and 2.5SY‐L (bottom) line. (b) Collection sites across Europe of the six populations that contributed to the “S” starting population. (c) A brief description of the multigeneration crossing scheme used to cross the six populations in (b) to generate the mixed “S” population used for experimental evolution. (d) Overview of traits assayed in each phenotyping session (8 in total, labelled P1 through P8). Inclusion of lines and assay diets in a phenotyping is diagrammed using filled (included) vs. unfilled boxes (not included). Briefly, the first column of boxes indicates the experimental evolution lines included, while the second, third and fourth columns indicate the assay diets on which they were assessed (key in inset box). In all cases, both the early (E) and late (L) reproducing lines were included. Thus, for example, in P4 (generation 10 and 5 of E and L lines, respectively), the 0.25‐E, 0.25‐L, 1‐E, 1‐L, 2.5‐E and 2.5‐L lines were all included (first column all filled), but only assayed under the 1.0 assay diet. It is noteworthy that there is a relatively large generation gap between sessions P1 through P5 and P6 through P8

Here, we combine variation in available nutrition during development with classical selection for early or late reproduction during adulthood in a single fully factorial EE design, using the fruit fly, *Drosophila melanogaster* (Figure 1a). Empirical work suggests that the two selection regimes might exert opposing selection pressures, which will have to be integrated into the life history. For example, adaptation to a poor quality diet generally selects for faster development coupled with smaller adult size and decreased fecundity (Bochdanovits & Jong, 2003; Kolss et al., 2009), whereas longer lifespan (the typical response to selection on increased age at reproduction) is generally correlated with longer developmental time and larger size (Lints, 1978; Economos, 1980; Promislow, 1993; Khazaeli, Van Voorhies, & Curtsinger, 2005, but see Zwaan, Bijlsma, & Hoekstra, 1991).

Our experimental design allows us to address several fundamental problems, including the question of whether adaptation to environmental variation in each stage occurs independently. For example, will a lower calorie developmental diet constrain the ability to extend lifespan in response to selection on age at reproduction, or will lifespan extension be achieved at the expense of other traits? To address this issue, we assess the evolutionary responses of several life‐history traits. These include larval survival, developmental time and adult weight, all of which have previously been found to evolve in response to larval acquisition (e.g., Bochdanovits & Jong, 2003; Kolss et al., 2009), as well as adult lifespan and fecundity, the two traits that commonly trade off in response to selection on age at reproduction (Luckinbill et al., 1984; Rose, 1984). Furthermore, we assay traits over multiple generations and in multiple larval dietary environments to gain insight into the temporal dynamics of evolution, and the evolution of phenotypic plasticity.

## 2 MATERIALS AND METHODS

### 2.1 Design of the experimental evolution experiment

We combined three levels of larval diet (0.25, 1.0 and 2.5) with two ages at reproduction (early and late) in a fully factorial design (Figure 1a, inset). The larval diets differed only in sugar and yeast content with the 0.25 and 2.5 diets containing 25% and 250% as much sugar and yeast as the 1.0 diet, respectively (Table S1). These diets were chosen to fall within the range typically applied in studies of diet and life history in *D. melanogaster* (Lee et al., 2008; Magwere, Chapman, & Partridge, 2004; Zajitschek et al., 2016). The early (E) and late (L) reproducing populations had generation times of 14 and 28 days, respectively; thus, adults laid eggs for the subsequent generation roughly 2–4 days post‐eclosion in the early (E) lines, and 16–18 days post‐eclosion in the late (L) lines (Figure 1a). For each combination of larval diet and age at reproduction, we established four independent replicate lines (three larval diets × two ages at reproduction × four replicate lines = 24 lines total; Figure 1a, inset). All lines were maintained on the 1.0 diet as adults both in the course of evolution and in all experiments. We refer to the EE lines by their larval diet (0.25, 1.0, 2.5), their age at reproduction (E, L) or the combination of the two (0.25‐E, 0.25‐L, 1‐E, 1‐L, 2.5‐E and 2.5‐L) throughout. Since the diet and age‐at‐reproduction conditions of the 1‐E lines mimic those of our standard laboratory maintenance regime, their responses can be considered representative of the baseline response in terms of both plasticity and the evolutionary response across generations. Lines were maintained throughout under standard laboratory conditions (25°C, 65% relative air humidity, 12 hr:12 hr light : dark cycle).

### 2.2 Generating the starting population and initiating experimental evolution

To ensure ample standing genetic variation, the EE populations were derived from six populations of flies collected along a latitudinal gradient across Europe (Figure 1b). These populations were maintained in the laboratory for 40 generations to allow for laboratory adaptation and then combined into a single panmictic, genetically diverse baseline population, the starting (“S”) population, using a multigeneration crossing scheme (Figure 1c; see May, Doroszuk, & Zwaan, 2015 for full details of the crossing scheme). This scheme was employed to minimize linkage disequilibrium and to ensure equal contributions of the component populations to the final “S” population. After crossing, the “S” population was maintained under standard laboratory conditions for a further 10 generations at a population size of ~4000 individuals.

To initiate EE, eggs were collected from the “S” population into large glass bottles (500 ml volume) filled with 65 ml of the respective larval diets. Two bottles of ~1000–2000 eggs were collected per replicate line and allowed to develop to adulthood. For each larval diet, four lines were randomly assigned to the early (E) and late (L) reproduction regimes. Ten days after egg laying (Monday), we transferred all newly eclosed adults into fresh bottles containing the 1.0 diet. Populations were then transferred to fresh bottles of 1.0 medium every Monday, Wednesday and Friday until their respective ages at reproduction. Since larval diet affected developmental time, not all adults from all lines had emerged by day 10, so on days 12 and 14 any newly eclosed adults from the developmental bottles were added to the adult population bottles to mitigate truncation selection on developmental time (Figure 1a). Very few flies eclosed after day 12.

The day before populations reached their respective ages at reproduction, 1/16 of a teaspoon of dry yeast (Fermipan Red Instant dry baker's yeast) was added to each bottle to stimulate reproduction. The following day females were transferred to fresh bottles containing their evolutionary larval diets and allowed to lay eggs. A test tube cap containing dry yeast mixed with water was suspended in the bottle and removed when egg laying was complete so as not to modify yeast levels in the developmental diet. To control egg densities, bottles were visually inspected, and adult flies were removed from bottles when egg density was between ~1000 and 2000 eggs, typically over a period of two to four hours. Every generation, both replicate bottles within a line were mixed. Overall, population size was ~2000 to 4000 adult flies per replicate line over the course of EE.

### 2.3 Assessing changes in life‐history traits over the course of evolution

We measured four key life‐history traits: egg‐to‐adult development time, mated female fecundity, mated lifespan and adult wet weight. We assayed these traits across eight independent phenotyping sessions, ranging from the beginning of EE up to generations 38 and 19 for E and L lines, respectively. Figure 1d provides an overview of each phenotyping session (P1‐P8), including the elapsed generations of EE, the lines included, the traits measured and the larval conditions under which flies were raised (i.e., assay environment). We deliberately chose larval diets that had negligible effects on larval survival to avoid population bottlenecks and strong viability selection. Larval survival ranged from 80 to 95% across evolutionary larval diets and assay conditions in all but one phenotyping session (Figure S1) and did not show any systematic variation across selection regimes (Table 1 and Figure S1), suggesting that larval survival was not under selection.

**Table 1:** Summary of GLMMs (chi‐square statistics, degrees of freedom, and their significance) for the effect of assay diet on larval survival, developmental time, lifespan and fecundity on 1‐E lines across phenotyping sessions. Where there was a significant effect of assay diet (i.e., plasticity for the response to assay diet), we report the outcomes of pairwise post hoc comparisons between assay diets (*p*‐values). Where several models were fit per trait, we indicate the subset analysed (Subset)

Phenotyping	Generation	Subset	Effect assay diet			Post hoc contrasts		
			Chi‐square	*df*	*p*‐value	P:C	P:R	R:C
Larval survival								
P3	7	—	1.59	2	0.45	—	—	—
P5	12	—	6.18	1	0.01	0.01	—	—
P6	30	—	0.42	2	0.81	—	—	—
P7	32	—	0.44	2	0.80	—	—	—
P8	38	—	5.46	2	0.07	—	—	—
Developmental time								
P3	7	—	1878.70	2	<0.0001	<0.0001	<0.0001	<0.0001
P5	12	—	1090.70	1	<0.0001	<0.0001	—	—
P6	30	—	2648.30	2	<0.0001	<0.0001	<0.0001	<0.0001
P7	32	—	2303.30	2	<0.0001	<0.0001	<0.0001	<0.0001
P8	38	—	4212.50	2	<0.0001	<0.0001	<0.0001	<0.0001
Fecundity								
P7	32	Early	12.904	1	<0.0001	0.0001	—	—
		Mid	570.12	1	<0.0001	<0.0001	—	—
		Late	392.35	1	<0.0001	<0.0001	—	—
P8	38	Early	251.46	2	<0.0001	<0.0001	<0.0001	0.79
		Mid	225.24	2	<0.0001	<0.0001	<0.0001	<0.0001
		Late	65.824	2	<0.0001	<0.0001	0.42	<0.0001
Lifespan								
P7	32	F	16.015	1	<0.0001	<0.0001	—	—
		M	32.831	1	<0.0001	<0.0001	—	—
P8	38	F	55.467	2	<0.0001	<0.0001	<0.0001	0.23
		M	46.134	2	<0.0001	<0.0001	<0.0001	0.45

Whenever possible we measured the responses to selection in all lines and used all three larval assay diets. However, the scale of our design imposed some logistical constraints. In some phenotyping sessions, we monitored the progress of adaptation on the 1.0 larval assay diet only, while in others, we raised larvae on all three diets. In all cases, we first allowed lines to develop for one generation on the 1.0 diet to avoid potential maternal effects. Larvae developed at a density of 70 eggs per vial, with 6 ml of food per vial. For each line, females laid eggs on agar petri dishes and eggs were then collected into vials and randomized across assay diets.

We assessed development time and survival from egg to adult in all eight phenotyping sessions (Figure 1d; *n *= 5 vials per combination of line and assay diet). We scored developmental time until no new flies emerged over a period of 48 hr and then summed across the resulting adults to obtain a measure of egg‐to‐adult survival (proportion viability). While using vials allowed easier standardization of egg densities and more accurate counting of eclosing adults, development took ~24 hr longer in vials than in the EE population bottles.

Mated lifespan and fecundity were assessed on the evolutionary larval diet and on the 1.0 diet. The size of this experiment necessitated two assays (Figure 1d): In the first round, we tested all lines adapted to 0.25 or 1.0 larval food on these two diets (P7), and in the second round, we tested all lines adapted to 1.0 and 2.5 larval diet across all three larval diets (P8). The 1.0 lines served as a reference to facilitate comparisons between the 0.25 and 2.5 lines and to monitor consistency of responses across both assays. For mated lifespan, we housed flies at a density of three males and three females per vial (*n *= 10 vials per combination of line and larval diet). Flies were transferred to fresh vials, and survival was scored every Monday, Wednesday and Friday.

Mated fecundity was measured over three time spans: early (days 2–4 of adulthood), late (days 18–21) and post‐selection (days 25–28) with the early and late time points overlapping the ages at reproduction of the E and L lines. In the first assay (P7), we maintained a single male–female pair per vial (*n *= 15 vials per line and larval food combination), while in the second assay (P8), we maintained two males and two females per vial (*n *= 10 vials per line and larval food combination). Eggs were allowed to develop to adulthood, and emerging adults were counted to score fecundity. Sperm depletion was prevented by replacing dead males with reserve males from the same experimental conditions.

Wet weight of adult males and females raised on the 1.0 assay diet was obtained in generations 144 and 73 of the E and L lines, respectively. All 24 EE populations were reared in small bottles (200 ml) with 25 ml 1.0 food at a density of 600–800 eggs per bottle (Clancy & Kennington, 2001). After eclosion, males and females were housed together until weighing (i.e., they were mated). Weight was measured at two time points chosen to mimic the conditions of the EE procedure: 14 days after egg laying (~4–5 days after emergence) and 28 days after egg laying (~18–19 days after emergence). The weight of the flies was measured on an ultramicro balance (Sartorius Cubis Ultramicro Balance MSE) using batches consisting of two flies each (*n *= 10). Prior to the assay, all populations were first reared for two generations on 1.0 medium. This assay was performed later than the other life‐history assays; however, the results were consistent with an interim measurement made on a subset of the lines at ER generation 100 and LR generation 50 (data not shown).

### 2.4 Statistical analysis

All statistical analyses were performed in R version 3.2.0 (R Development Core Team, 2005). We fitted a separate model for each trait within each phenotyping session. In each model, we included evolutionary dietary regime, age at reproduction, assay diet, sex (where applicable) and their interactions as explanatory variables. We used mixed‐effects models to accommodate the random effect of replicate line nested within a selection regime. Both developmental time and mated longevity were analysed using mixed‐effects Cox regression (proportional hazards) models (coxme package; Therneau, 2015), while larval survival and fecundity were analysed with generalized linear mixed models (GLMM) with binomial and Poisson error distributions, respectively (lme4 package; Bates, Maechler, Bolker, & Walker, 2015). Weight was analysed using a linear mixed‐effects model with a normal distribution. In the statistical tables (see below), we report the χ^2^ values of the effect of each factor in the full model as obtained by analysis of deviance (car package; Fox & Weisberg, 2010). We performed further model simplification by sequentially dropping nonsignificant terms from the model and using a χ^2^ test to compare models. To control for multiple comparisons, we applied the sequential Holm–Bonferroni correction method to each fitted model (Holm, 1979) and the Tukey range test for all post hoc comparisons among means (Tukey, 1949).

For each trait assessed across multiple assay diets, we fitted an additional model for the 1‐E lines alone (unselected control lines) to determine the baseline plastic response. An inconsistent response of the 1‐E lines across generations might indicate that the trait in question is sensitive to slight differences in developmental conditions; in this case, differences among lines might be highly dependent on variations in assay conditions and may thus not reflect robust evolutionary responses (cf. Ackermann et al. 2001).

## 3 RESULTS

### 3.1 Developmental time depends on selection regime by assay diet interactions

**Figure 2:**
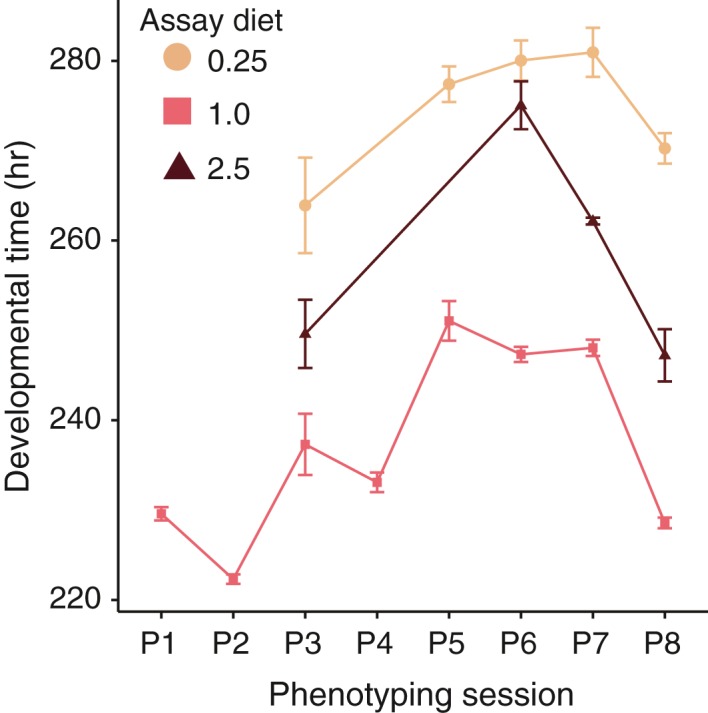
Developmental time from egg to adult (y‐axis) across phenotyping sessions (x‐axis) by assay diet (0.25: beige square; 1.0: pink circle; 2.5: purple triangle). Not all phenotyping sessions included all three assay diets. Each point represents taking the mean of the average developmental time for each of the lines included in the assay. For example, if all 24 lines were included in the assay, the mean developmental time was calculated per line, and then, the mean and standard error of these 24 values was calculated

**Figure 3:**
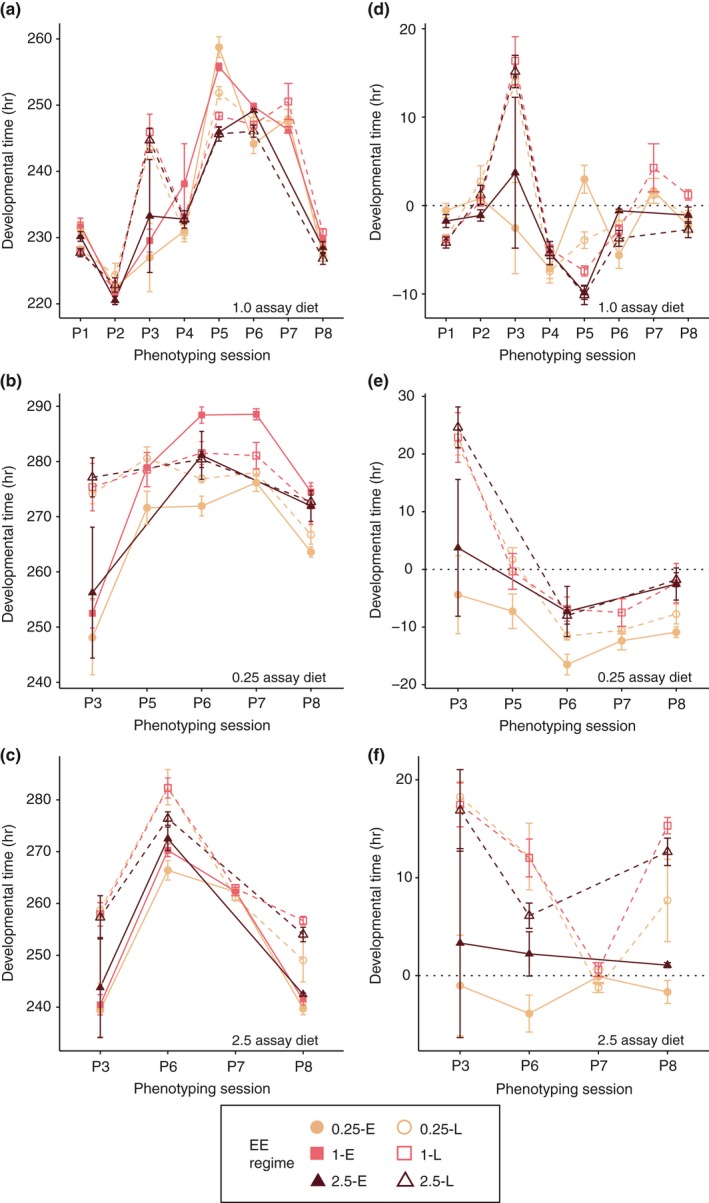
Developmental time from egg to adult (y‐axis) across phenotyping sessions (x‐axis) for the 1.0 assay diet (a,d), the 0.25 assay diet (b,e) and the 2.5 assay diet (c,f). (a‐c) represent the observed developmental times, while (d‐f) are the developmental times relative to the mean of the 1‐E lines. Each point represents taking the mean and standard error of the average developmental time for each of the four lines

Over the course of evolution, assay diet was the most important factor influencing developmental time (Table 2; Figure 2). The 0.25 and, to a lesser extent, 2.5 assay diet consistently increased developmental time relative to the 1.0 diet across all EE lines and phenotyping sessions (Figure 2 *p *< 0.00001 in all but one contrast). However, it is noteworthy that the mean duration of development on each of the three assay diets fluctuated greatly across phenotyping sessions (Figure 2). Such variation is not uncommon in repeated measures of developmental time (e.g., Zwaan, Bijlsma, & Hoekstra, 1995). To account for this, we plotted both the absolute values of developmental time per assay diet across phenotyping assays (Figure 3a–c) and relative to the mean of the 1‐E lines (Figure 3d–f).

**Table 2:** Summary of GLMMs (chi‐square values) for the effect of assay diet (A), evolutionary dietary regime (D) and evolutionary age at reproduction (R) on larval survival and developmental time across phenotyping sessions

Phenotyping	EE diet (D)	EE repro (R)	Assay diet (A)	D*R	D*A	R*A	D*R*A
Larval survival							
P1	0.26	0.05	—	0.24	—	—	—
P2	0.30	0.85	—	0.37	—	—	—
P3	0.71	0.02	6.6	0.57	1.50	0.00	0.66
P4	0.35	0.01	1.24	—	—	—	—
P5	13.06**	3.94	0.17	0.32	25.24***	0.46	0.85
P6	5.23.	2.49	42.69***	2.73	5.99	38.86***	12.91
P7	0.00	0.00	3.23	0.12	3.02	0.74	0.51
P8	1.45	0.04	8.07	0.62	2.68	0.43	3.66
Developmental time							
P1	2.07	27.58***	—	0.96	—	—	—
P2	4.14	0.63	—	0.68	—	—	—
P3	0.46	15.74***	20617.4***	0.34	140.61***	64.27***	72.31***
P4	3.86	2	—	0.97	—	—	—
P5	0.18	4.30	4830.06***	2.44	25.13***	244.81***	41.63***
P6	8.06	2.02	12746.57***	11.57**	76.11***	311.47***	48.51***
P7	6.90	0.18	8506.83***	1.06	22.29***	34.18***	78.39***
P8	7.64	13.36***	23285***	1.21	181.40***	758.70***	46.53***

Significance is indicated by * =*p *< 0.05, **=*p* < 0.01, ***=*p* < 0.001.

In the early generations of EE (P1–P5), no consistent changes in developmental time were observed (Figure 3). However, from P6 (E and L generations 30 and 15, respectively) onwards a consistent three‐way interaction emerged between evolutionary larval diet, age at reproduction and assay diet for the 0.25‐E and 0.25‐L lines (Table 2; Figure 3). Both sets of lines evolved substantially more rapid development on the 0.25 assay diet as compared to the 1‐E lines (Figure 3b,d). For the 0.25‐E lines, this effect was already present in P5 (P5 through to P8; all *p*‐values <0.001), while for the 0.25‐L lines, it became apparent from P6 onwards (P6 through to P8; all *p*‐values <0.001; Figure 3b,e), although there was a trend for the magnitude of the effect to be smaller for the 0.25‐L than 0.25‐E lines (P5: *p *< 0.0001; P6: *p* = <0.01; P7: *p* = 0.33; P8: *p* = 0.26). By contrast, on the 1.0 and 2.5 assay diets, the responses of the 0.25‐E and 0.25‐L lines were not consistent across phenotypings (Figure 3a,c,d,f). Relative to the strength and consistency of the response of the 0.25‐E and 0.25‐L lines, the 1‐L and 2.5‐E and 2.5‐L lines did not show substantial or consistent changes in developmental time, suggesting that these regimes did not impose strong selection on the length of development. These data suggest that the increased development time on 0.25 led to a large selective pressure to decrease this trait in order to be able to reproduce in time. This explains the difference between 0.25‐E and 0.25‐L lines. The fact that 0.25‐L lines also showed a decrease in development time but, to a lesser extent, indicates a potential effect of diet itself.

### 3.2 Selection on age at reproduction increases lifespan across dietary selection regimes

**Figure 4:**
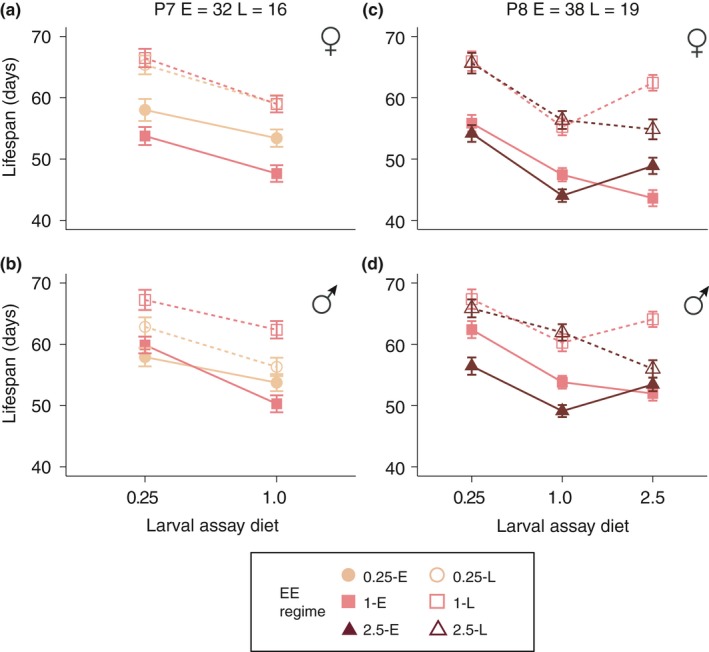
Lifespan (y‐axis) across assay diets (x‐axis) and phenotyping sessions P7 (a, b) and P8 (c, d) for females (a, c) and males (b, d). Lifespan is expressed as days from adult eclosion. All error bars are standard errors of the mean across replicate lines

We found that selection for increased age at reproduction increased lifespan in all lines and across all assay diets (Figure 4; all *p*‐values < 0.03). However, the magnitude of the effect was dependent on sex and the evolutionary dietary regime for 0.25 lines and evolutionary dietary regime and assay diet for 2.5 lines, suggesting that adaptation to different levels of larval acquisition can modify the response to selection on lifespan (Table 3).

**Table 3:** Summary of GLMMs (chi‐square values) for the effect of assay diet (A), evolutionary dietary regime (D) and evolutionary age at reproduction (R) on lifespan across phenotyping sessions

Factor	Phenotyping	
	P7	P8
Evo diet (D)	0.26	8.45*
Evo repro (R)	15.66***	28.63***
Assay diet (A)	3557.81***	3761.66***
Sex (S)	0.01	46.47***
D*R	4.13*	0.49
D*A	2.08	9.73**
R*A	0.02	0.55
S*D	12.69***	10.34**
S*R	1.63	16.94***
S*A	0.00	2.62
D*R*A	0.27	20.99***
R*A*S	0.44	0.59
S*D*R	4.43*	0.01
S*D*A	1.68	0.01
S*D*R*A	1.59	0.22

Significance of chi‐square values are indicated by * =*p* < 0.05, **=*p* < 0.01, ***=*p* < 0.001.

For the 0.25‐E and 0.25‐L lines, males and females had inverse responses to selection relative to the 1‐E and 1‐L lines (Table 3; Figure 4a,b). In females, the lifespan of the 0.25‐L lines was indistinguishable from that of the 1‐L lines (*p* = 0.94), while lifespan was increased in the 0.25‐E lines (*p *< 0.0001; Figure 4a). In males, the exact inverse response was observed: While the 0.25‐E and 1‐E lines had similar lifespans (*p *= 0.96), the lifespan extension of 0.25‐L lines was less than that of the 1‐L lines (*p *= 0.01; Figure 4b). These effects were consistent across both the 0.25 and 1.0 assay diets.

For the 2.5‐E and 2.5‐L lines, lifespan evolved in a similar manner in both sexes, but was dependent on assay diet (Table 3 and Figure 4c,d). Under 0.25 and 1.0 assay conditions, the lifespan of the 2.5L lines did not differ from the 1‐L lines (*p *> 0.2 for both conditions); however, under 2.5 assay conditions, 2.5‐L flies evolved significantly shorter lifespans than 1‐L lines in males (*p *= 0.002, Figure 4d) and nearly significantly shorter lifespans in females (*p *= 0.08, Figure 4c), indicating that the evolved phenotype is only expressed under the evolutionary relevant condition. The 2.5E lines showed an inverse pattern: Lifespan on the 0.25 and 1.0 assay diets was generally higher for 1‐E lines than for 2.5‐E lines, whereas males and females of the 2.5‐E lines outlived 1‐E flies on the 2.5 assay diet (Figure 4c,d; males: on 0.25 and 1.0 diet 1‐E > 2.5‐E, *p *= 0.003 and 0.004, respectively; under 2.5 assay conditions 1‐E = 2.5‐E, *p *= 0.66. Females: on 0.25 assay diet 1‐E = 2.5‐E, *p *= 0.42; on 1.0 assay diet 1‐E > 2.5‐E lines, *p *= 0.02; and under 2.5 assay diet 1‐E < 2.5‐E lines, *p *= 0.01).

### 3.3 Fecundity is highly variable across phenotyping sessions

**Figure 5:**
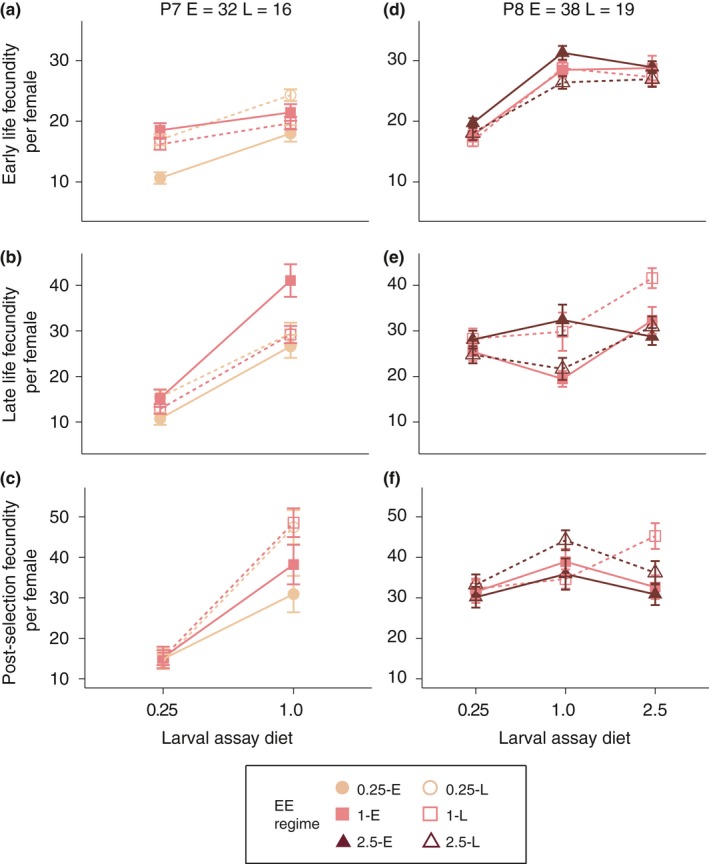
Reaction norms of realized early (a,d), late (b,e) and post‐selection (c,f) female fecundity (y‐axis) across assay diets (x‐axis) and phenotyping sessions P7 (a:c) and P8 (d:f). All error bars are standard errors of the mean across replicate lines

**Figure 6:**
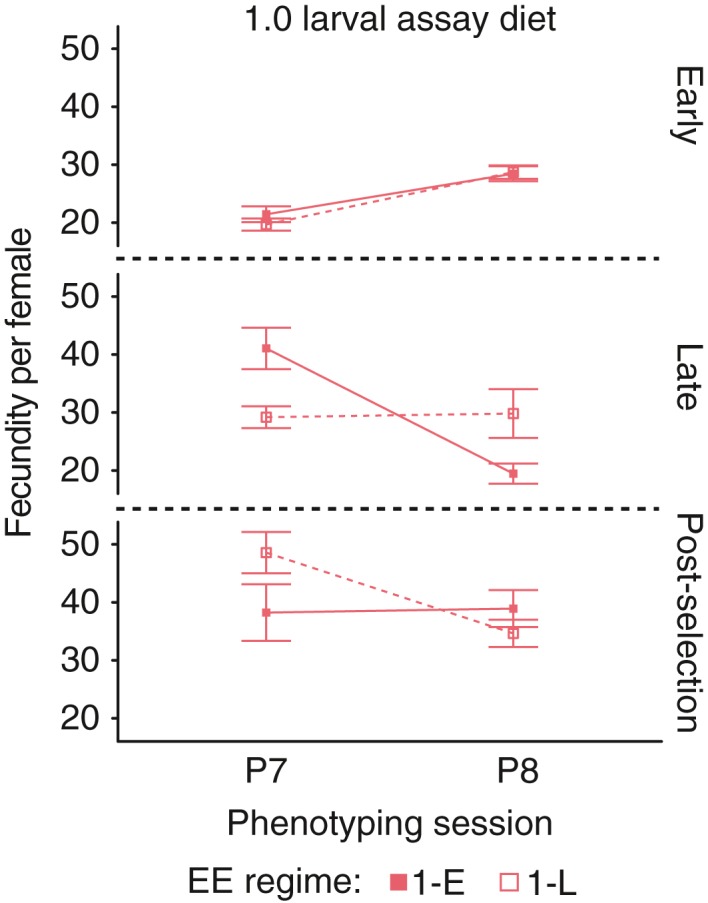
Inconsistencies in fecundity of 1.0 lines across phenotyping sessions. All error bars are standard errors of the mean across replicate lines

Because it was not possible to measure lifespan and fecundity for all lines at the same time, we used the 1‐E and 1‐L lines as a standard across the two replicate phenotyping sessions (see Materials and Methods). For mated fecundity, the plastic response of the 1‐E lines to assay diet differed between the two phenotyping sessions (Table 1, Figure 5). In the first phenotyping (P7), 1‐E flies raised on the 0.25 assay diet had lower fecundity than those raised on the 1.0 assay diet at all three ages (Figure 5a‐c; all *p*‐values <0.001). In the second assay (P8), the same effect was observed at early and post‐selection ages (all *p*‐values <0.001), but reversed at the late reproduction time point (*p *< 0.001; Figure 5d,f). Furthermore, the difference between the 1‐E and 1‐L lines on the 1.0 assay diet was also inconsistent between assays P7 and P8 (Figure 6). In P7, the E lines reproduced more than the L lines at the “Mid” time point and less at the “Late” time point, while in P8, the opposite pattern was observed (Figure 6, both *p*‐values <0.003). Thus, while the GLMMs indicated that fecundity at all ages was affected by interactions between dietary regime, age at reproduction regime and assay conditions (Table 4), the lack of consistency of the 1‐E and 1‐L lines hampers the interpretation of the evolutionary significance of these effects.

**Table 4:** Summary of GLMMs (chi‐square values) for the effect of assay diet (A), evolutionary dietary regime (D) and evolutionary age at reproduction (R) on fecundity at early, mid and late ages across phenotyping sessions

Phenotyping	Age	Evo diet (D)	Evo repro (R)	Assay diet (A)	D*R	D*A	R*A	D*R*A
P7	Early	1.00	1.85	176.80***	4.45	35.26***	1.18	5.47
	Mid	4.69	0.49	1383.33***	8.14*	8.02*	20.00***	0.72
	Late	1.5	7.5*	2154.05***	0.7135	1.84	76.34***	7.62*
P8	Early	0.20	1.60	892.25***	0.77	9.05	0.49	8.05
	Mid	0.62	0.89	364.66***	10.20**	87.46***	43.04***	132.38***
	Late	0.29	7.54*	204.34***	0.934	84.75***	64.578***	90.37***

Significance of chi‐square values are indicated by * =*p* < 0.05, **=*p* < 0.01, ***=*p* < 0.001.

### 3.4 Adult weight

**Figure 7:**
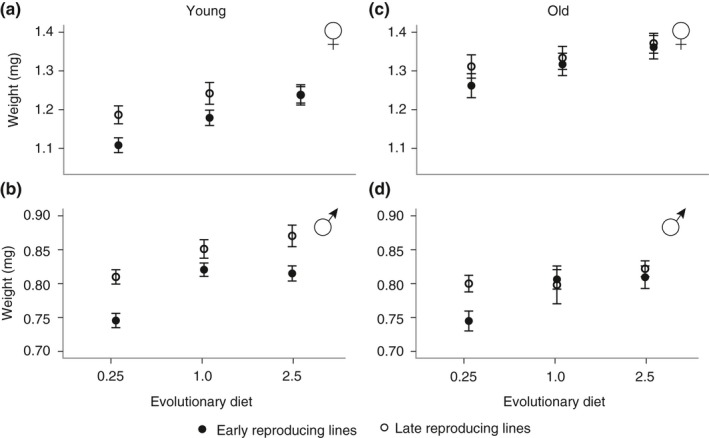
Body weight (mg) of female (a,c) and male (b,d) flies raised on the 1.0 assay diet at young (~4–5 days old) and old (~18–19 days old) ages. All error bars are standard errors of the mean across replicate lines

Adult weight evolved in response to the selection regimes in a sex‐ and age‐dependent manner (Figure 7a‐d). The largest effects of the EE regimes occurred in young flies (4–5 days post‐eclosion): In both sexes, adaptation to later ages at reproduction led to higher adult weight (females: F_1,24_ = 8.4, *p *=* *0.01; Figure 7a; males: F_1,24_ = 43.7, p=<0.0001; Figure 7b), and adaptation to the 0.25 larval diet decreased adult weight relative to 1.0 and 2.5 adapted lines (females:F_2,24_ = 10.9, *p *=* *0.001; Figure 7a; males: F_2,24_ = 29.6, p=<0.0001; Figure 7b; all pairwise *p*‐values <0.01). In females, there was a marginal interaction between age at reproduction and evolutionary dietary regime (F_2,24_ = 2.7, *p *=* *0.09, Figure 7a). While the 0.25‐L and 1‐L lines both evolved increased weight relative to the 0.25‐E and 1‐E lines, the weight of the 2.5‐E and 2.5‐L lines did not differ (2.5‐L = 2.5‐E, *p *=* *1.0). At 18–19 days post‐eclosion, the effects of evolutionary regime became much smaller and differed between the sexes. In males, the effect of EE regime was largely absent, except in 0.25‐E lines, which continued to weigh less than all other lines (all pairwise *p*‐values <0.003; Figure 7d), while in females, only evolutionary dietary regime remained significant (F_2,24_ = 9.1, *p *=* *0.001, Figure 7c), with weight increasing with increasing evolutionary larval diet (all pairwise *p*‐values <0.05). We also found large effects that were independent of the evolutionary regimes: Males weighed less than females (Sex: F_1,936_ = 11644, *p*=<0.0001) and, while females gained weight with age, males tended to lose or maintain the same weight (Sex x Age: F_1,936_ = 314.3, *p*=<0.0001; Figure 7). The fact that the 0.25‐E lines weigh less at both time points indicates that this might be due to body size effects, a trait limited by developmental time. This is corroborated by the fact that 2.5E and 2.5L lines did not differ in body size, indicating that development time differences under less constricted dietary condition do not have to lead to a decrease in body weight.

## 4 DISCUSSION

### 4.1 Adaptive responses reflect the influence of both selection regimes

Increased age at reproduction extended lifespan across all evolutionary dietary regimes and in both sexes (Luckinbill et al., 1984; Partridge & Fowler, 1992; Rose, 1984), while selection on the 0.25 larval diet resulted in faster development (Figure 3b,e), decreased adult weight (Figure 7) and potentially lower fecundity (Figure 5a‐c), again, in keeping with previous univariate selection experiments (Kolss et al., 2009; Kristensen et al., 2011). However, in both cases, we found that the addition of a second regime modified the magnitude of the responses. Thus, the extent of the increase in lifespan imposed by selection for later age at reproduction was dependent on dietary regime (Figure 4), and conversely, the changes in weight, length of development and potentially fecundity seen in the 0.25‐E lines were modified by adding selection for late reproduction. The fact that both selection regimes only exert a modifying effect suggests that genetic constraints are far from absolute, and degrees of freedom for evolution of combinations of trait values exist. This in turn indicates that it is unlikely that a few genetic variant underlie these adaptations (Jha et al., 2015). Furthermore, it also shows that the physiological constraints, such as those imposed by the 0.25 diet on lifespan extension, are only partially limiting and that these constraints can be resolved, potentially by the evolution of third party traits, such as fecundity and body weight.

### 4.2 Fecundity: significant but inconsistent responses

Previous EE designs selecting on later age at reproduction also found inconsistent responses of fecundity across generations (Leroi, Chen, & Rose, 1994), or marked sensitivity to environmental variation (Leroi, Chippindale, & Rose, 1994). However, we observed strongly significant effects of both age at reproduction and evolutionary dietary regime in both phenotyping sessions (Table 4). For example, 0.25‐E lines appeared to have decreased fecundity relative to 0.25‐L, 1‐E and 1‐L lines at all ages (Figure 5a‐c), a response that is consistent with their lower body weight and faster development (Figures 3b,e and 7). Given the large replication of our design (i.e., independent replicate populations per EE treatment), it is plausible that these responses represent adaptive responses to poor nutrition.

However, what hampers firm conclusions about fecundity are the inconsistent phenotypes of the 1.0 line females across the P7 and P8 sessions (Figure 6). The slightly different assay conditions between the two phenotyping sessions (one male and one female per vial in P7 vs. two females and two males per vial in P8) present one potential cause as females are known to adjust their fecundity based on density (e.g., Barker, 1973; this study compares the difference between vial densities of 5 and 50 females or more). Slight changes in environmental conditions (e.g., note the considerably faster development in P8 relative to P7; Figure 2) might also have affected overall patterns of fecundity. Our results indicate that while certain traits evolve in a more predictable manner, such as development time and lifespan, fecundity might depend more on individual condition and environment. Furthermore, the existence of both positive and negative pleiotropy between fecundity and lifespan (van den Heuvel et al., 2017) might hamper the formation of clear predictions about the expected mean values of fecundity in such long‐lasting evolutionary trajectories.

### 4.3 Does adult body size drive patterns of life‐history adaptation?

In many studies, body size correlates positively with developmental time, lifespan and fecundity (see above and Robertson, 1957; Hillesheim & Stearns, 1992; Honěk, 1993; Zwaan et al., 1995; Prasad, Shakara, Anitha, Rajamani, & Joshi, 2001). Our results also showed such correlations; for instance, selection for late life reproduction extended lifespan and increased adult weight for males and females alike. However, these correlations are unlikely to constrain the evolution of the life‐history adaptations, but will rather modulate them. For instance, while selection for late reproduction consistently increased lifespan for the 0.25 lines, these lines also sped up their development and reduced their weight relative to the 1 and 2.5 lines. Furthermore, the fact that the differences in body weight between early and late life populations were large in early life, but disappeared later in life (at the time of actual selection for the late lines) for 1 and 2.5 but not 0.25 lines (Figure 7), suggests that body size evolved for a different reason in these lines relative to the 0.25 lines. For instance, increased body size as a response to late life reproduction in the 0.25 lines may serve to increase fecundity in the face of decreased adult weight as an adaptive response to the larval nutritional condition, while in the 1 and 2.5 lines increased body size it may be related to increasing lifespan.

## 5 CONCLUSIONS

Our results suggests that adaptation during one life stage may be contingent on the selection pressures experienced in other stages and that adaptation to two different selection pressures can lead to different life‐history strategies to those found when adapting to only one selection pressure at a time. In particular, the dependence of lifespan extension on evolutionary developmental diet suggests that developmental acquisition can be an important factor influencing longevity. However, the consistent increased of development time and lifespan in late age‐at‐reproduction lines indicates that both genetic and physiological constraints are not absolute. Genetically, this can be explained by the fact that many loci with small effects determine these life‐history traits, while multitrait evolution can resolve or modify certain physiological constraints. While there is still relatively little empirical work on adaptation to multiple or opposing selection pressures (but see: Lankau, 2007; Tarwater & Beissinger, 2013), their prevalence in nature means that a better understanding can further our understanding of evolution under natural conditions (reviewed in Schluter et al., 1991). Indeed, the idea that opposing selection pressures constrain trait evolution is one of the hypotheses put forward to explain why, despite strong consistent directional selection on many traits, there is often little change in trait means across generations in natural populations (Kingsolver & Diamond, 2011; Merilä, Sheldon, & Kruuk, 2001; Siepielski, DiBattista, Evans, & Carlson, 2011). Given that multiple selection pressures are likely the norm rather than the exception in nature, our findings suggest that trade‐offs should be considered not only between traits within an organism, but also between adaptive responses to differing selection pressures.
