# Supplementary material for: Adaptation to developmental diet influences the response to selection on age at reproduction in the fruit fly
Source: J Evol Biol. 2019 Feb 27;32(5):425–37. doi: 10.1111/jeb.13425 (PMC6850652; doi:10.1111/jeb.13425)
Supplement: Supplementary file 2 [file JEB-32-425-s002.docx]

Supplementary Table 1: Diet composition per liter of water

| Diet composition | 0.25 | 1.0 | 2.5 |
| --- | --- | --- | --- |
| Yeast* | 17.5g | 70g | 175g |
| Sugar † | 25g | 100g | 250g |
| Agar | 20g | 20g | 20g |
| Nipagin solution | 15mL | 15mL | 15mL |
| Propionic acid | 3mL | 3mL | 3mL |

*Fermipan Red Label instant yeast

†Suiker Unie Granulated Sugar Extra Fine
